# Women with Recurrent Miscarriage Have Decreased Expression of 25-Hydroxyvitamin D_3_-1α-Hydroxylase by the Fetal-Maternal Interface

**DOI:** 10.1371/journal.pone.0165589

**Published:** 2016-12-29

**Authors:** Li-qin Wang, Xiao-ting Yan, Chun-fang Yan, Xin-wen Zhang, Ling-yun Hui, Mingzhan Xue, Xue-wen Yu

**Affiliations:** 1 Department of Obstetrics and Gynecology in First Affiliated Hospital of Xi’an Jiaotong University, Xi’an, China; 2 Nursing Department in Xi’an Medical College, Xi’an, China; 3 Reproductive Center in Fourth Hospital of Xi'an, Xi’an, China; 4 Laboratory Department in First Affiliated Hospital of Xi’an Jiaotong University, Xi’an, China; 5 Clinical Sciences Research Lab, Translational Medicine Section, Division of Biomedical Sciences, Warwick Medical School, University of Warwick, University Hospital, Coventry, United Kingdom; Xavier Bichat Medical School, INSERM-CNRS - Université Paris Diderot, FRANCE

## Abstract

**Background:**

Effects of vitamin D deficiency in pregnancy have been associated with some adverse pregnancy outcomes. The 25-hydroxyvitamin D_3_-1α-hydroxylase (CYP27B1) is integral to the vitamin D metabolic pathway. The enzyme catalyzes localized conversion of pro-hormone 25-hydroxyvitamin D_3_ to active 1,25-dihydroxyvitamin D_3_. Our aim was to investigate the expression of CYP27B1 at the fetal-maternal interface in the first trimester pregnancy and to determine whether CYP27B1 was associated with recurrent miscarriage (RM).

**Methods:**

Expressions of CYP27B1 mRNA and protein in villi and decidua from 20 women undergoing primary miscarriage, 20 women with RM and 20 women with normal pregnancy were evaluated by western blot, and quantitative real-time PCR. The co-localization of CYP27B1 and certain cytokines including IL-10, IFN-γ, TNF-α, and IL-2 expression were examined using immunohistochemistry and confocal microscopy.

**Results:**

Women with RM had a significantly lower expression of CYP27B1 mRNA and protein in villous and decidual tissues compared with the normal pregnant women (P = 0.000 in villus, P = 0.002 in decidua for mRNA; P = 0.036 in villus, P = 0.007 in decidua for protein.). Compared with the normal pregnancy, immunostaining for CYP27B1 was significantly decreased in villous trophoblasts and decidual glandular epithelial cells in RM women. No significant differences in the localization of CYP27B1, IL-10, IFN-γ, TNF-α, and IL-2 expression were identified between the normal pregnant and RM women.

**Conclusions:**

Women with RM have a lower level of CYP27B1 expression in chorionic villi and decidua compared with normal pregnant women, suggesting that reduced CYP27B1 expression may be associated with RM. The consistent localization of CYP27B1 and IL-10, IFN-γ, TNF-α, and IL-2 expression in villous and decidual tissues suggests the importance of the local production of 1,25(OH)_2_D_3_ at the fetal-maternal interface to regulate cytokine responses.

## Introduction

The incidence of recurrent miscarriages (RM) is approximately 1–3% of all couples of reproductive age [1]. Several mechanisms have previously been described for the pathogenesis of RM, including chromosomal anomalies, hormonal problems, uterine abnormalities, infections, and autoimmune disorders. The etiology of approximately 50% of RM is not fully understood. Immunological mechanisms have been proposed to explain at least some of these cases of RM [2–3]. The active form of vitamin D, 1,25 dihydroxyvitamin D_3_ (1,25(OH_2_D_3_), is a lipid-soluble hormone that has well-established classic effects on bone metabolism and mineral homeostasis. Except its calciotropic function, recent investigations suggested the 1,25(OH)_2_D_3_ has potent immunomodulatory effects on various tissues [4–6]. The discovery of immunomodulatory functions of 1,25(OH)_2_D_3_ has led to increased interest in its role in pregnancy[7]. Consistently, the enzyme that catalyzes the synthesis of 1,25(OH)_2_D_3_, 25-hydroxyvitamin D_3_-1α-hydroxylase(CYP27B1) has a widespread distribution in various tissues. Viganòet al. reported that CYP27B1 is expressed in human endometrial stromal cells independently of both proliferative and secretory phase of the menstrual cycle and the first trimester decidual cells, its expression is up-regulated in early pregnant versus normal cycling endometria of both phases [8]. In a recent research conducted by Tavakoli et al. [9], endometrial stromal cells and whole endometrial cells from the healthy controls and women with RM express CYP27B1 and are found to have the comparable capacity to produce the active form of vitamin D_3_. The presence of the enzyme in cycling endometrium, whole endometrial cells from women with RM, and its up-regulation in first trimester decidua, support the possibility that this hormone may be involved in some mechanisms of pregnancy establishment or maintenance. In addition, a study *in vitro* from Díaz et al. [10] has shown that calcitriol inhibits significantly the tumor necrosis factor-α (TNF-α)-induced expression of interferon γ (IFN-γ) in cultured human trophoblasts, a process that conceivably reduces the likelihood of pregnancy-associated disorders such as miscarriage [2–3]. Based on previous studies [5,11–12], we hypothesize that RM in first trimester pregnancy may be associated with aberrant expression of the CYP27B1 at the fetal-maternal interface. However, molecular analysis of the CYP27B1 at the fetal-maternal interface in the first trimester pregnancy and women with RM has drawn very limited attention. Thus, we have studied expression and localization of CYP27B1 at the fetal-maternal interface of women in the first trimester pregnancy and analyzed the association of CYP27B1 expression and RM. Furthermore, we investigated expressed localization of TNF-α, IL-2, IFN-γ, and IL-10 at the fetal-maternal interface.

## Material and Methods

### Research subjects

This was an observational study. Forty pregnant women with unexplained vaginal bleeding at 7–10 weeks of gestation, who had been referred to First Affiliated Hospital of Xi’an Jiaotong University, Shaanxi Province, China, between October 2013 and October 2014, after confirmed embryo demise by transvaginal ultrasound, were enrolled in early miscarriage (EM) group. All the women had vaginal bleeding for the first time in the prior 48 h. An intact gestational sac, absence of fetal heartbeat matched for gestational age, confirmed the diagnosis of EM. Of them, 20 having at least two previous consecutive pregnancy losses at early gestation were as RM group. 20 women undergoing primary pregnancy loss were as primary miscarriage group (PM). All women were younger than 35, had regular menstrual cycle and none had uterine anomalies, chromosomal abnormalities, thyroid dysfunction, diabetes mellitus, autoimmune disorders, infection with rubella, toxoplasma, cytomegalovirus and herpes virus, and received hormones for at least 3 months. Their gestational age, based on the last menstrual period, was confirmed by ultrasound examination. 20 age-matched women who had at least one healthy live birth undergoing elective termination of normal pregnancies between 7 and 10 weeks of gestation were included in the control group. Women in the control, PM and RM groups did not differ significantly in average age (28.9±3.1y, 28.8±2.7y, 29.5±2.4y, F = 0.373, P = 0.69) and average gestational age (54.8±4.4d, 56.4±4.2d, 58.1±4.7d, P = 2.688, P = 0.08).

Chorionic villous and decidual samples were collected by curettage on the day of or the second day of embryo demise diagnosis and on day of undergoing therapeutic termination of pregnancy. For RNA and protein extraction, villous and decidual samples were snap-frozen in liquid nitrogen and stored at -80°C. For immunohistochemistry and confocal laser scanning microscopy (CLSM), villous and decidual samples were routinely processed and frozen at -70°C.

Patients were informed that tissue samples would be used for research purposes and they gave a written consent. The study was approved by the Ethics Committee of the First Affiliated Hospital of Xi’an Jiaotong University (2 March 2012).

### Assessment of decidual and chorionic villous CYP27B1 by Streptavidin-peroxidase Immunohistochemistry

All tissue samples (20 controls and 20 RM) were washed in 0.9% sterilized sodium chloride as soon as they were removed from uterus. After fixed in 4% buffered paraformaldehyde and treated with 25% saccharose solution, the samples were frozen at -70°C. Cryosections of 5-μm thickness were prepared using a tissue microtome (Microm HM500 O, Microm International GmbH Walldorf, Germany). At least one cryosection from each case was stained with haematoxylin and eosin (H&E) to allow morphological assessment. Cryosections of the villus and decidua were analyzed using a streptavidin-peroxidase immunohistochemistry kit (Zymed Laboratories, Inc., San Francisco, CA, USA). Briefly, the cryosections were immersed in 1:10 diluted hydrogen peroxide (stock H_2_O_2_ concentration 30%) with PBS for 10 minutes and blocked with normal goat serum for 20 min at room temperature, followed by anti-CYP27B1 polyclonal antibody (1:400; Abcam Inc., Cambridge, MA) for 2 h at 37°C. A peroxidase-conjugated goat anti-rabbit polyclonal was used as the secondary antibody. The primary antibody was omitted as the negative controls. Digital images were acquired using a section microscope scanner (Leica MP SCN400, Germany). The five fields in each section were analysed. 20 cells of each field (in total 100 cells for per sample) were estimated using software Image Proplus 6.0. The average gray-scale value from 100 cells was obtained. To further exclude operator bias, observations were performed on coded samples in a blinded manner following the same procedure.

### Co-localization of decidual and chorionic villous CYP27B1 with TNF-α, IL-2,IFN-γ and IL-10 by double immunofluorescence

Immunofluorescence staining (6 villous and decidual tissues including 3 controls and 3 RM women) was used to localize and compare the distribution of CYP27B1 and TNF-α, IL-2, IFN-γ and IL-10. The 5-μm-thick cryosections were air-dried and immersed in a buffer (30% H_2_O_2_: distilled water = 1:10) for 10 min at room temperature. After blocking with normal goat serum for 20 min at room temperature, the sections were incubated with anti-CYP27B1 polyclonal antibody diluted at 1:100 for overnight at 4°C, followed by incubation with antibody including diluting anti-TNF-α (1:500), IL-2 (1:50), IFN-γ (1:500), and IL-10 (1:100) (Boster Biological Technology, Ltd., China) for 10 h at 4°C, respectively. Then, the sections were incubated with carbocyanine 3 (Cy3)-conjugated and AlexaFlur 488-conjugated secondary antibody (Boster Biological Technology, Ltd., China) for 2 h at 37°C. The omitted primary antibody was used as negative control. Confocal laser scanning microscopy (CLSM) (TCS SP2, Leica Co., Germany) was used for evaluation of morphology and the level of protein expression. To further exclude operator bias, observations were performed on coded samples in a blinded fashion. The fluorescence excitation was provided by a 488/560 nm argon laser beam and emission was 535/650 nm for FITC/Cy3. The images were analyzed using the software Image Plus (Leica, Germany).

### Assessment of decidual and villous CYP27B1 by western blot

200 mg of villous and decidual samples were taken from collected whole tissue. After lysis of the samples in 1mL of lysis buffer containing 1mM of phenylmethanesulfonyl fluoride, the tissue lysate was centrifuged at 12,000×g for 20 min at 4°C in a microcentrifuge. Then, transferring the supernatant (i.e. protein sample) to a fresh tube kept on ice, removing small volume (50 μL) of supernatant to perform a protein assay by a commercial protein assay kit (BCA Protein Assay Kit, Beijing Tiangen Co., China), the remaining volume of supernatant was added 1/4 volume of 5×Sample Buffer, boiled for 5 minutes at 100°C, and aliquoted. The protein (20 μg for per sample) samples were resolved by sodium dodecyl sulfate-polyacrylamide gel electrophoresis (SDS-PAGE) and transferred to polyvinylidene fluoride (PVDF) membranes (Millipore, USA). After blocking with 8% milk for 2 h at room temperature, the membranes were incubated with anti-CYP27B1 polyclonal antibody (Abcam Inc., Cambridge, MA) overnight at 4°C, followed by peroxidase-conjugated goat anti-rabbit IgG for 2 h at 37°C. For the protein load control, anti-β-actin rabbit monoclonal antibodies (Beijing Biosynthesis Biotec. CO., LTD., China) were used. The image was acquired using darkroom development techniques for chemiluminesence. Results were expressed as the ratio of signal intensity of CYP27B1 bands to β-actin (the loading control).

### Assessment of decidual and villous CYP27B1 mRNA by quantitative real-time PCR assay

Real-time RT-quantitative polymerase chain reaction (RT-qPCR) analysis was performed on total RNA isolated from snap-frozen villous and decidual tissues. Briefly, total RNA was isolated from villous and decidual tissues with the Mini BEST Universal RNA Extraction Kit (TaKaRa, Dalian, China). Complementary DNA (cDNA) was then synthesized from the RNA (1μg from each sample) using PrimeScript^TM^ RTMaster Mix (TaKaRa, Dalian, China). PCR reactions in a reaction mixture of the SYBR^@^ Premix Ex Taq^TM^II (Tli RNaseH Plus) (TaKaRa, Dalian, China) were carried out in triplicates using SYBR Green and they were performed using the CFX96 Touch™ Real-Time PCR Detection System (Bio-Rad Laboratories, California, USA). Human β-actin (ACTB) gene was used as reference gene. Specific oligonucleotide primers for amplification of CYP27B1 and ACTB were used. The sequences of primers were as follows: forward 5’-CCAAAGCCAAAGGGAAGAGA-3’, reverse 5’-AAGGCGGTGGTCAAGGAA-3’ for CYP27B1, and forward 5’- TGGCACCCAGCACAATGAA-3’, reverse 5’-CTAAGTCATAGTCCGCCTAGAAGCA-3’for β-actin. The PCR conditions were 30 s at 95°C, followed by 39 cycles at 95°C for 5 s and 60°C for 30 s. The results were analyzed using the comparative ΔΔCt method. For each experimental sample, 2^-ΔΔCt^ was calculated, and the data were reported as relative expression levels.

### Statistical analysis

SPSS-PC+ for Windows was used. Fluorescence intensity and greyscale value were expressed as the mean ±SEM. Statistical significance was determined using one-way ANOVA and the multiple comparisons in ANOVA. A P<0.05 was considered significant.

## Results

### Decidual and villous CYP27B1 mRNA by RT-qPCR

**Fig 1:**
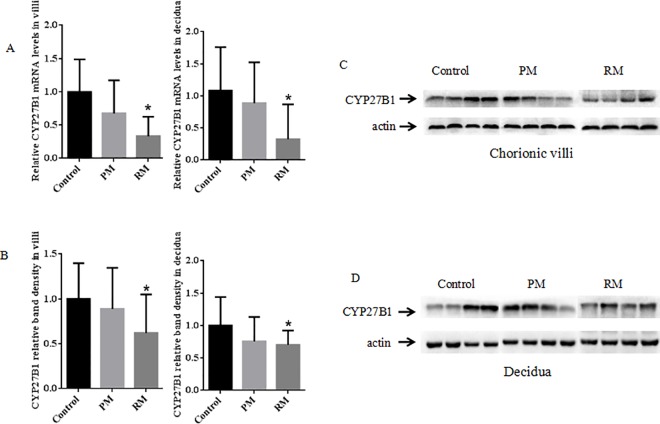
CYP27B1 expression in chorionic villous and decidual tissues. (A) RT-qPCR analysis of CYP27B1 mRNA in villous and decidual tissues obtained from normal pregnant women, PM and RM in the first trimester pregnancy. Left: chorionic villus; Right: decidua. (B) CYP27B1 protein expression in chorionic villous and decidual tissues from normal pregnant women, PM and RM. Left: chorionic villus; Right: decidua. Western blots profile of total homogenate of villous (C) and decidual tissues (D). Four representative cases of early pregnancy from normal pregnant women, PM and RM were shown. Detection of β-actin was used for protein load control.

The expression of CYP27B1 gene transcription was detected in the villous and decidual samples from RM, PM and control groups by RT-qPCR. Results of the experiments indicated that levels of CYP27B1 mRNA were similar in villous and decidual tissues but were significantly different among RM, PM and controls (F = 10.534, P = 0.000; F = 6.741, P = 0.003). It showed a decrease of approximately 32% and 67% in CYP27B1 mRNA expression in chorionic villi and 11% and 67% in decidua in the PM group and RM group compared with the control group, respectively (Fig 1A). The expression levels of CYP27B1 mRNA in villous tissues were 0.40±0.35 and 1.20±0.58, respectively, in RM and control group, which showed a significant difference (P = 0.000). However, CYP27B1 expression in decidual tissues in RM group was not only significantly down-regulated compared to its expression in control group (0.44±0.73 vs 1.35±0.84, P = 0.002), but also decreased in comparison with PM group (1.20±0.86, P = 0.007) (Table 1).

**Table 1:** Comparison of CYP27B1 mRNA in villous and decidual tissues in the control, PM, and RM group (mean±SEM).

Tissues	n	Control	PM	RM	F	P
		(n = 18)	(n = 18)	(n = 18)		
Villous	54	1.20±0.58	0.81±0.60	0.40±0.35*	10.534	0.000
Decidual	54	1.35±0.84	1.20±0.86	0.44±0.73*^,^^#^	6.741	0.003

*Significantly lower compared to the RM group and control group (p = 0.000 and 0.002, respectively).

^#^Significantly lower compared to the RM group and PM group (p = 0.007).

### Decidual and villous CYP27B1protein by western blotting

Western blot analysis was used to assess the total amount of CYP27B1 protein in the chorionic villi and decidua of pregnant women in PM, RM and control groups. A 57-kD western blot bands that corresponded to CYP27B1 protein was detected in samples of chorionic villi and decidua. It showed that there were significantly different in CYP27B1 protein level in villous and decidual tissues among PM, RM and controls (F = 4.110, P = 0.022; F = 3.901, P = 0.026). Densitometric analysis of western blots showed a reduction of about 30% and 24% in CYP27B1 protein expression in decidua and 38% and 12% in villi from RM and PM versus normal decidua and villous tissues from controls (Fig 1B). The CYP27B1 expression levels in decidua and villi from RM were significantly lower than that from controls (P = 0.007, P = 0.036; Table 2). CYP27B1 protein levels were normalized to β-actin (Fig 1C and 1D).

**Table 2:** Comparison of CYP27B1 protein expression in villous and decidual tissues in the control, PM, and RM group.

Tissues	n	Control	PM	RM	F	P
		(n = 20)	(n = 20)	(n = 20)		
Villous	60	1.45±0.58	1.28±0.66	0.90±0.62*	4.110	0.022
Decidual	60	1.58±0.70	1.20±0.59	1.11±0.35*	3.901	0.026

*Significantly lower compared to the RM group and control group (p = 0.007 and 0.036, respectively).

Gray-scale values are represented as mean±SEM.

### Decidual and villous CYP27B1 protein by immunohistochemistry

**Fig 2:**
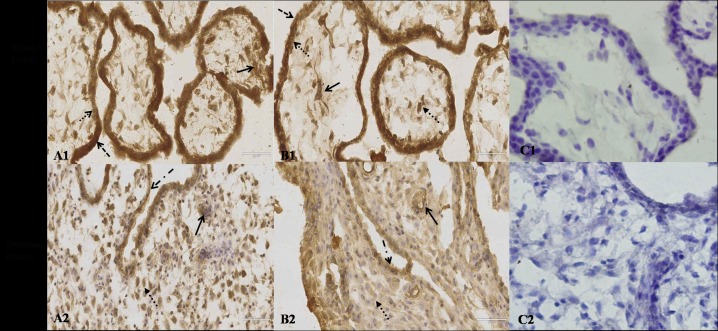
Immunohistochemistry for CYP27B1: A1, Normal villous tissue, A2, villous tissue with RM, B1, normal decidual tissue, B2, decidual tissue with RM, C1, villous tissue without primary antibody, and C2, decidual tissue without primary antibody. The arrows show the immunostaining cells. (Scale bar, 73 μm)

In order to determine the cellular localization of CYP27B1 in villous and decidual tissue, its expression was also evaluated in RM and control group by immunohistochemistry. Comparable immunostaining for CYP27B1 was present in the villous and decidual tissues of both RM and control group. Immunopositive cells were diffusely distributed throughout the villi and decidua including villous stromal cells, syncytiotrophoblast cells, cytotrophoblast cells, vascular endothelial cells, decidual stromal cells, and glandular epithelial cells. Furthermore, CYP27B1 was strongly expressed in trophoblasts (gray-scale value: 0.45±0.05 for cytotrophoblast, 0.46 ± 0.06 for syncytiotrophoblast) in normal villous tissues, but markedly reduced in trophoblasts (gray-scale value: 0.39±0.05 for cytotrophoblast, 0.40 ± 0.05 for syncytiotrophoblast) in RM’s villous tissues (t = 3.052, P = 0.005 for cytotrophoblast; t = 2.667, p = 0.012 for syncytiotrophoblast) (Fig 2A1 and 2B1). CYP27A1 was also strongly expressed in decidual glandular epithelial cells (gray-scale value: 0.45±0.05) in normal early pregnancy, and its expression was decreased in RM’s decidual glandular epithelial cells (gray-scale value: 0.35±0.06, t = 2.328, P = 0.028.) (Fig 2A2 and 2B2). But CYP27B1 expression in villous and decidual tissues was not significantly different between PM and control group (data not shown).

### Co-localization of CYP27B1 and IL-10, IFN-γ, TNF-α, IL-2 protein by double immunofluorescence

**Fig 3:**
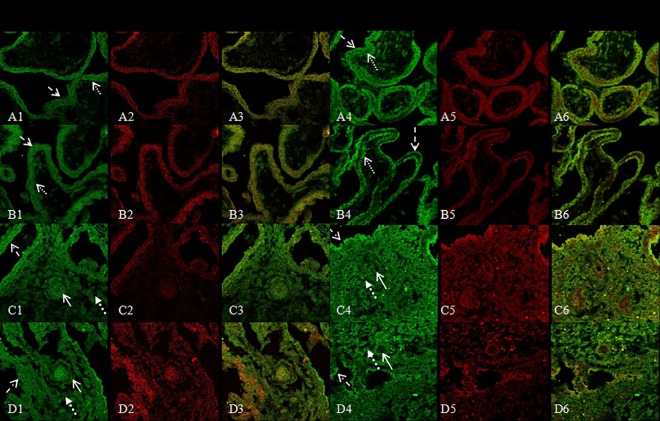
CLSM for CYP27B1 and IFN-γ, IL-10: A1,B1,C1,D1,A4,B4,C4,D4,CYP27B1, A2,B2,C2,D2, IFN-γ, and A3,B3,C3,D3, overlayed CYP27B1 and IFN-γ; A5,B5,C5,D5,IL-10, and A6,B6,C6,D6, overlayed CYP27B1 and IL-10 in A1-A6, normal villous tissue, B1-B6, villous tissue with RM, C1-C6, normal decidual tissue, and D1-D6, decidual tissue with RM. Green color indicates positive staining for CYP27B1 protein; Red color indicates IFN-γ, IL-10. The arrows show the immunostaining cells. (CLSM×400)

**Fig 4:**
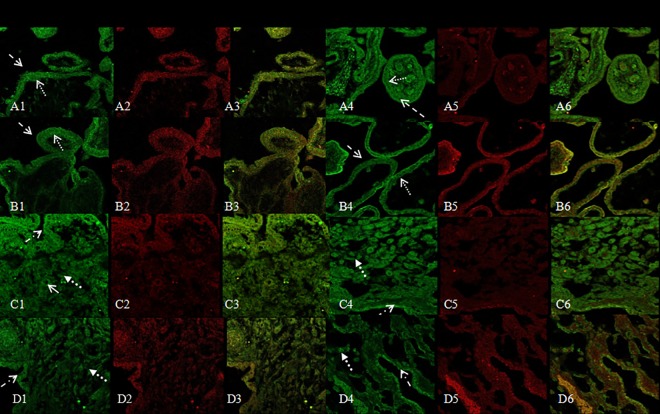
CLSM for CYP27B1 and TNF-α, IL-2: A1,B1,C1,D1,A4,B4,C4,D4,CYP27B1, A2,B2,C2,D2, TNF-α, and A3,B3,C3,D3, overlayed CYP27B1 and TNF-α; A5,B5,C5,D5,IL-2, and A6,B6,C6,D6, overlayed CYP27B1 and IL-2 in A1-A6, normal villous tissue, B1-B6, villous tissue with RM, C1-C6, normal decidual tissue, and D1-D6 decidual tissue with RM. Green color indicates positive staining for CYP27B1 protein; Red color indicates TNF-α, IL-2. The arrows show the immunostaining cells. (CLSM×400)

Immunofluorescence staining was used to localize and compare the distribution of CYP27B1 and IL-10, IFN-γ, TNF-α and IL-2 in villous and decidual tissues. Immunostaining results showed that CYP27B1 and IL-10, IFN-γ, TNF-α, and IL-2 were co-expressed in villous and decidual tissues from normal pregnant and RM women. Like CYP27B1, in normal villous and decidual tissues, IL-10 was expressed in trophoblasts and decidual glandular epithelial cells, but IL-10 immunostaining decreased in trophoblasts and decidual glandular epithelial cells in RM group (Fig 3). CYP27B1 and IFN-γ, TNF-α, and IL-2 were also co-expressed in trophoblasts in normal villous tissues, and immunostaining for IFN-γ, TNF-α, and IL-2 were increased in villous tissues from RM (Figs 3 and 4). In decidual tissues, CYP27B1, IFN-γ, TNF-α, and IL-2 were visualized in glandular epithelial cells, vascular endothelial cells and stromal cells. Immunostaining for IFN-γ, TNF-α, and IL-2 were increased in decidual glandular epithelial cells in RM samples (Figs 3 and 4). No significant differences in the localization of CYP27B1, IL-10, IFN-γ, TNF-α, and IL-2 expression were identified between the normal pregnant and RM women.

## Discussion

The human placenta expresses vitamin D receptor (VDR), retinoid X receptor (RXR), CYP27B1 and 24-hydroxylase [13]. In agreement with these findings, we found that CYP27B1 was expressed in both villous and decidual tissues in the first trimester pregnancy. Studies *in vitro* and *vivo* have shown that the CYP27B1 allows local conversion of pro-hormone 25-hydroxyvitamin D to active 1,25(OH)_2_D_3_ [14]. It is suggested that the CYP27B1 was a local regulator of levels of 1,25 (OH)_2_D_3_, and it was expressed in villous and decidual tissues in the first trimester pregnancy (in these data), meaning that villous and decidual tissues were an extrarenal site of vitamin D synthesis and vitamin D functioning target. In particular, villous and decidual samples from healthy pregnancies showed strong immunolocalization of CYP27B1 within villous trophoblast cells and decidual glandular epithelial cells, indicating an important role for the enzyme in the first trimester pregnancy.

Data presented here indicated that both mRNA and protein for CYP27B1 were abundantly expressed in villous and decidual tissues from the first trimester pregnancy, and significantly decreased in villous and decidual tissues from RM pregnancies compared to those from the healthy pregnancies. The relatively low level of CYP27B1 in villous and decidual tissues from RM pregnancies raised the possibility that the enzyme played a local role in embryo implantation. In female mice, both VDR and CYP27B1 knockout mice had significantly fewer viable fetuses in utero and present with absent corpora lutea [11]. Compared to CYP27B1^+/+^ placentas after vehicle-treated, CYP27B1 knockout placentas increased expression of Th1 cytokines and decreased expression of Th2 cytokines, which means that dysregulated vitamin D metabolism in placenta promotes aberrant immune responses [6].In the first trimester pregnancy, expression of CYP27B1 and synthesis of 1,25(OH)_2_D_3_ have much higher activity at the decidual tissue [15]. Women with higher vitamin D level in their serum and follicular fluid were more likely to achieve clinical pregnancy following *in vitro* fertilization [16–17]. The immunosuppressive effects of 1,25(OH)_2_D_3_ allowed proper trophoblast invasion of the uterus without triggering a maternal immune response [14]. The decidualization induced by 1,25(OH)_2_D_3_ contributed to implantation in the first trimester pregnancy [18]. Indeed, it had been suggested that 1,25(OH)_2_D_3_ synthesized by the fetal-maternal interface during pregnancy was not meant to function as a hormone, but as an immunomodulatory cytokine to prevent a fetus-versus-mother reaction and rejection of the fetus [19].

In order to understand local immunomodulatory effects of vitamin D at the fetal-maternal interface *in vivo*, we investigated expressive localization of CYP27B1 and TNF-α, IL-2, IFN-γ, IL-10 and compared their distribution at the fetal-maternal interface in the first trimester pregnancy. We found that CYP27B1 and TNF-α, IL-2, IFN-γ, IL-10 were co-expressed in villous trophoblast in normal pregnancy, but CYP27B1 and IL-10 markedly decreased and TNF-α, IL-2, and IFN-γ markedly increased in trophoblasts in villous tissues in RM pregnancy. The presence of CYP27B1 in trophoblasts indicated that local synthesis of 1,25(OH)_2_D_3_ had the potential influence on the local immune system. In primary cultured human cytotrophoblasts, 1,25(OH)_2_D_3_ inhibited expression of cytokines, such as TNF-α, IFN-γ, and increased expression of cAMP [15,20–22]. Similarly, impaired maternal 1,25(OH)_2_D_3_ during pregnancy influence the level of IL-10 in the maternal and cord blood. The level of IL-10 was lower in 1,25(OH)_2_D_3_ deficient women than in insufficient and sufficient pregnant women [23]. The evidences obtained in human and in rodent models showed increased production of proinflammatory cytokines such as IFN-γ, IL-2 and TNF-α was potentially harmful for pregnancy outcome, while predominance of anti-inflammatory cytokines was thought to be beneficial for pregnancy success [2]. Indeed, in early miscarriage high levels of plasma TNF-α, TNF-α receptor, IFN-γ and low levels of plasma IL-10 have been found, including TNF-α, TNF-α receptor, IL-10 at the fetal-maternal interface [24–29]. So, an adequate balance of the cytokine profile was necessary for successful pregnancy. Vitamin D is a key factor in regulation of the immune responses to balance pro/anti-inflammatory cytokines by regulation Vitamin D dependent cytokines expression at the fetal-maternal interface. CYP27B1, as an enzyme that catalyzes the synthesis of 1,25(OH)_2_D_3_, played an important role in controlling expression of 1,25(OH)_2_D_3_ at the fetal-maternal interface during pregnancy. The adequate expression of CYP27B1 at the fetal-maternal interface might prevent rejection of the implanted embryo.

In this study, we found that different levels of CYP27B1, TNF-α, IL-2, IFN-γ, and IL-10 in trophoblasts and decidual glandular epithelial cells from normal pregnancy and RM pregnancy. However, it was not clear whether increase of TNF-α, IL-2, and IFN-γ and decrease of IL-10 expression in RM were due to decreased expression of VDR. For the future, the expressions of these cytokines in the calcitriol-stimulated trophoblasts or decidual glandular epithelial cells with knockin or knockout CYP27B1 gene will be studied in animal model and cell model from normal pregnant women and RM women. These findings will support the results from *in vivo* studies.

In the present work, we focused only on CYP27B1 level at the fetal-maternal interface in the first trimester pregnancy during women with miscarriage and normal pregnant women. Although it is expected that CYP27B1 level plays an important role in fetal-maternal interface during the first trimester pregnancy, the impact of the fetal karyotype on CYP27B1 gene activity in the first trimester pregnancy miscarriage is not known. It is not clear whether reduced CYP27B1 level is associated with local immuneregulation of the fetal-maternal interface in RM. The precise molecular mechanisms by which CYP27B1 contributes to decidual and trophoblast functions leading to RM or fetal karyotype has impact on the placental and local target gene responses in the first trimester pregnancy miscarriage warrant further investigation.

Collectively, the data provide experimental evidence which links RM in humans with expression of CYP27B1 at the fetal-maternal interface in the first trimester pregnancy. Women with RM have a lower level of CYP27B1 expression in chorionic villi and decidua compared with normal pregnant women, suggesting that reduced CYP27B1 expression may be associated with RM. The relatively low levels of CYP27B1 at the fetal-maternal interface in RM women decrease the possibility that the enzyme plays a role in implantation process and a local immunological embryo-protection.In addition, CYP27B1 and TNF-α, IL-2, IFN-γ, and IL-10 are co-expressed in villous and decidual tissues in the first trimester pregnancy. These findings suggest the importance of the local production of 1,25(OH)_2_D_3_ at the fetal-maternal interface to control adverse immunological responses related to implantation. Future experiments should explore 1,25(OH)_2_D_3_ regulation of immunological responses induced by cytokines in embryo implantation.
